# Supplementary material for: Structural insight into Marburg virus nucleoprotein–RNA complex formation
Source: Nat Commun. 2022 Mar 4;13:1191. doi: 10.1038/s41467-022-28802-x (PMC8897395; doi:10.1038/s41467-022-28802-x)
Supplement: Supplementary file 1 — Supplementary Information [file 41467_2022_28802_MOESM1_ESM.pdf]

SUPPLEMENTARY INFORMATION

Structural insight into Marburg virus nucleoprotein-RNA complex formation

Yoko Fujita-Fujiharu[1,2,3], Yukihiko Sugita[1,2,4], Yuki Takamatsu[1,#], Kazuya Houri[1,2,3], Manabu Igarashi[5], Yukiko Muramoto[1,2,3], Masahiro Nakano[1,2,3], Yugo Tsunoda[1,2,3], Ichiro Taniguchi[6], Stephan Becker[7,8], Takeshi Noda[1,2,3*]

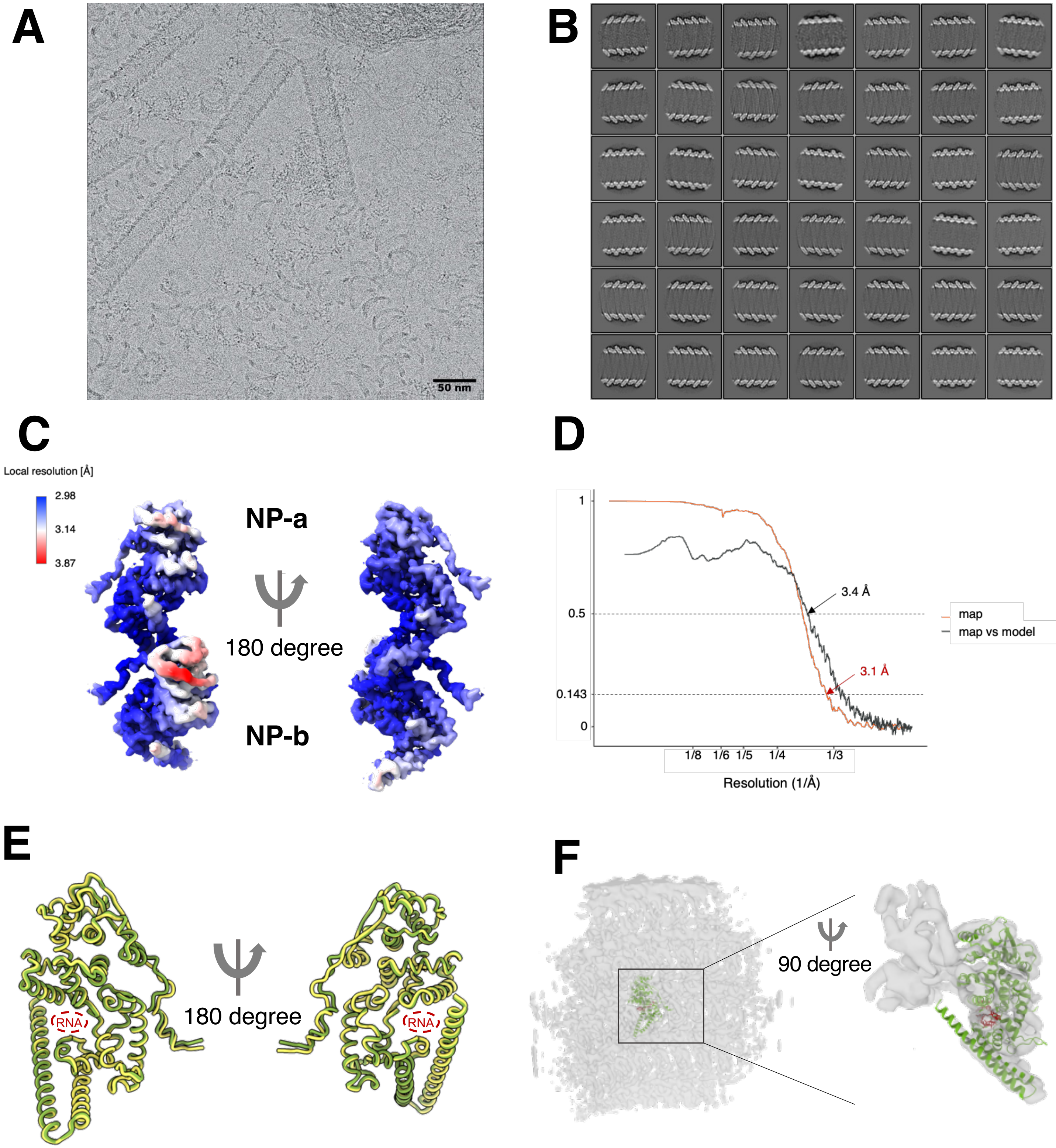

Supplementary Fig. 1: Cryo-EM image and single particle analysis of MARV NP−RNA complex.
(A) A representative cryo-electron micrograph of purified MARV NP−RNA complex. The helical NP−RNA structures were observed reproducibly.
(B) 2D class averages of MARV NP−RNA complex, selected for the following 3D classification.
(C) Local resolution map of the asymmetric subunits, NP-a and NP-b, in a MARV NP−RNA complex.
(D) Fourier Shell Correlation (FSC) curves from independently refined datasets. Red curve for overall map resolution of 3.1 angstrom (FSC = 0.143), which is validated by the cross-FSC between cryo-EM map and model-generated map (black, 3.4 angstrom at FSC = 0.5).
(E) Overall structure of MARV RNA-bound NP-a structure (green) superimposed with NP-b structure (yellow).
(F) RNA-bound NP-a structure (colored in light green) fitted into authentic MARV nucleocapsid EM map (EMD-3875, 2.5σ).

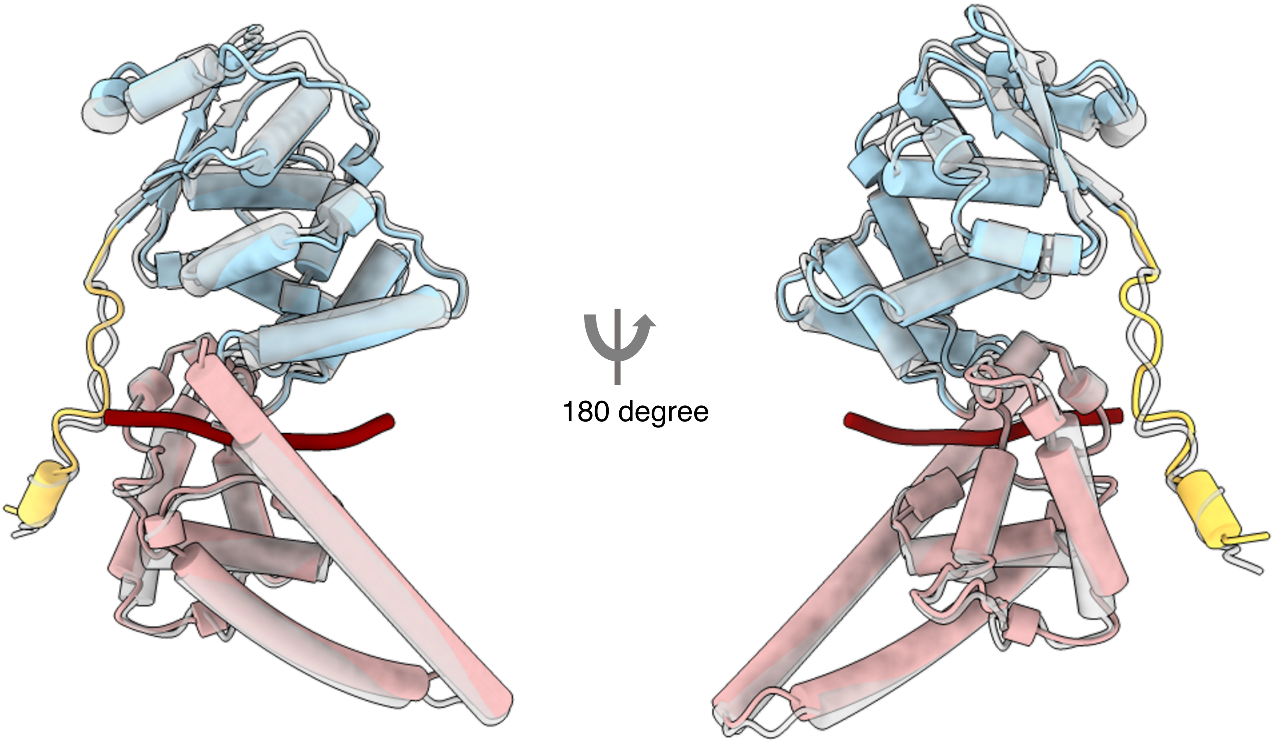

Supplementary Fig. 2: Comparison between EBOV and MARV NP−RNA complex unit structures. Overall structure of MARV NP−RNA complex (PDB-ID: 7F1M, colored as shown in Fig. 1a, from this study) is superimposed with EBOV NP−RNA complex (PDB-ID: 5Z9W, gray).

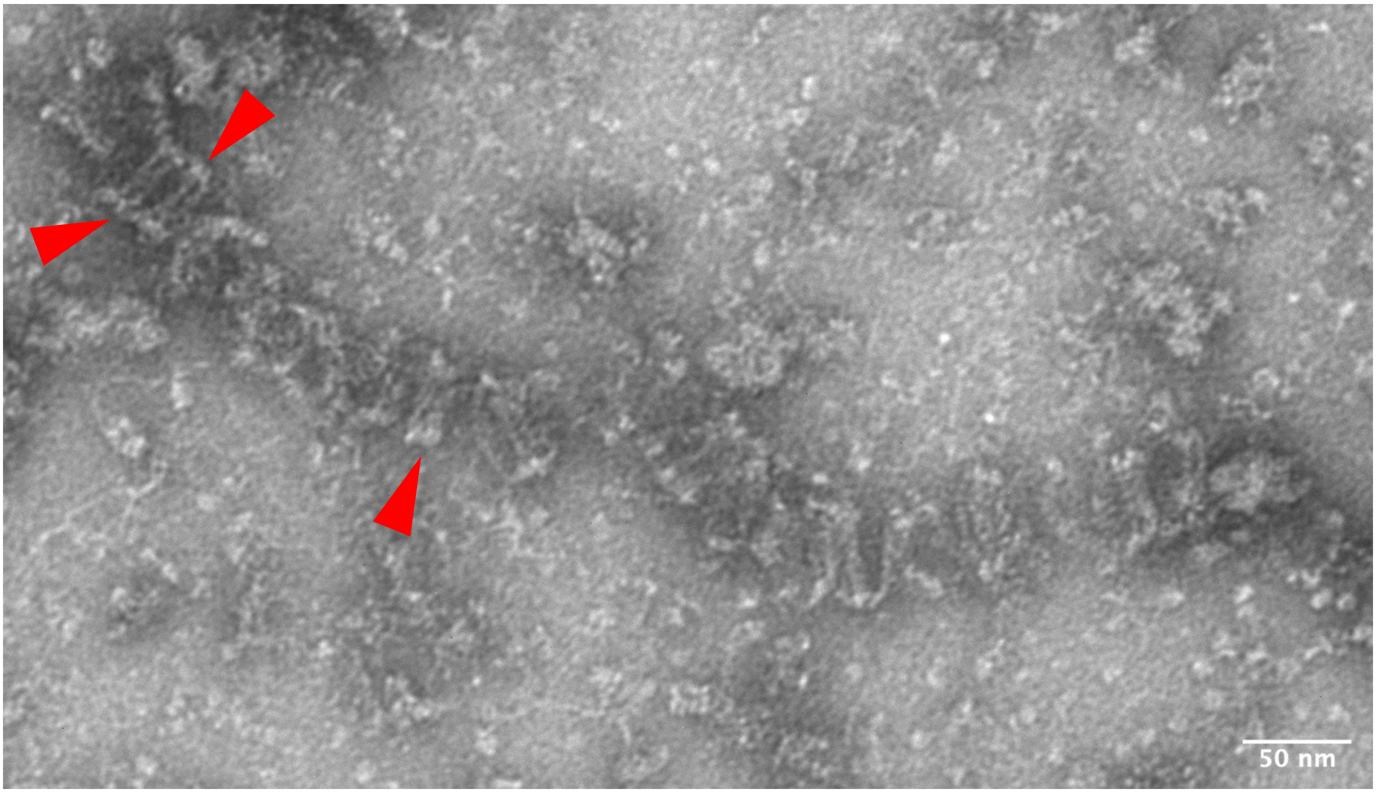

Supplementary Fig. 3: MARV full length NP−RNA complexes.
An image of the negatively stained MARV full length NP−RNA complexes purified from Expi 293F cells. Red arrow indicates a double-helical structure. The experiments were performed in duplicates (n = 2).

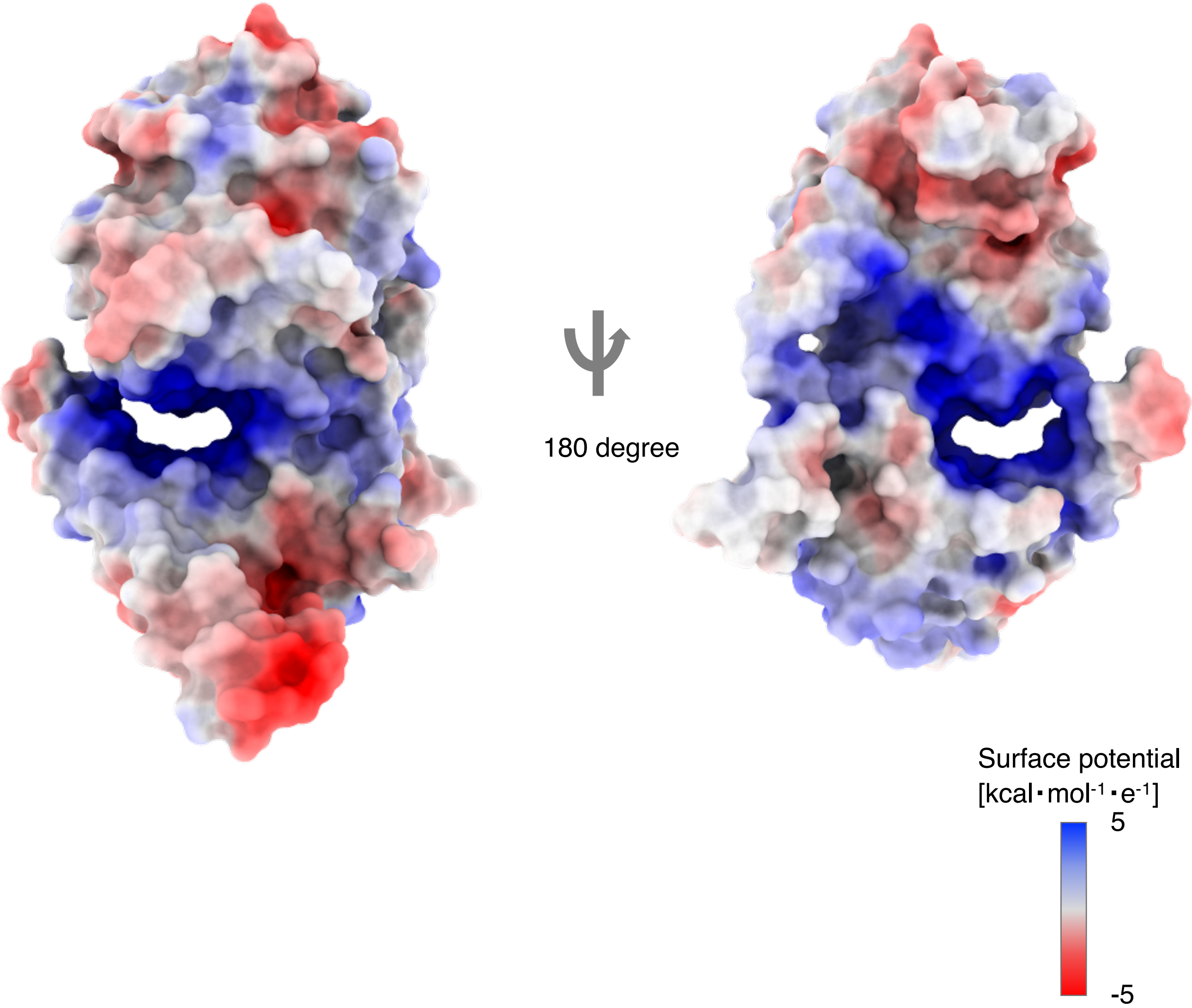

Supplementary Fig. 4: Electrostatic surface of MARV NP-a.
Electrostatic surface potential was calculated with the atomic model of our NP-a monomer using Delphi web-server[40,41]. Scale ranging from -5 (red) to +5 kcal mol^{-1} e^{-1} (blue).

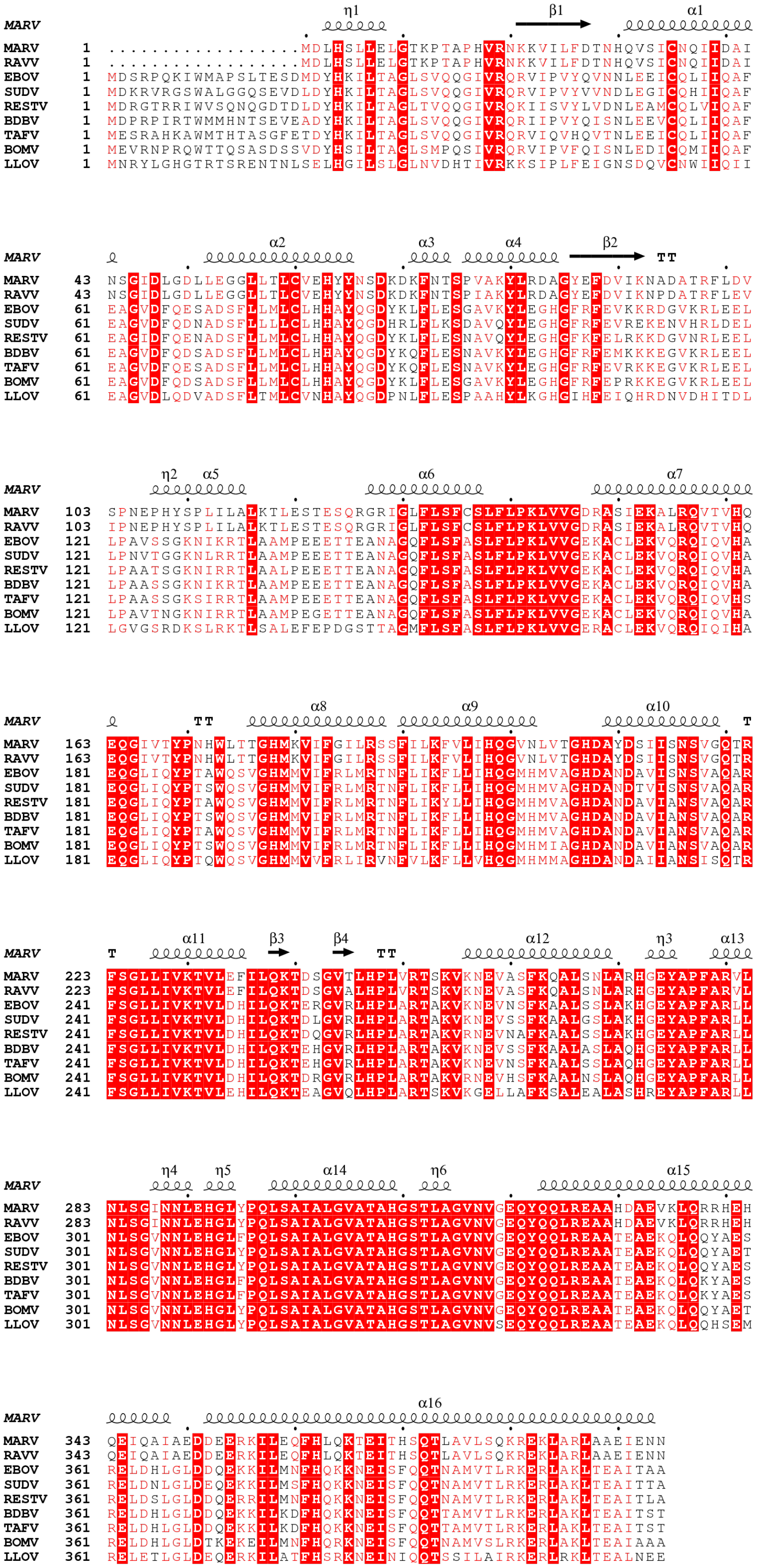

Supplementary Fig. 5: Secondary structure assignment of MARV NP-a aligned with filovirus NPs.

Secondary structure elements were annotated by phenix.secondary_structure_restraints[30]. The amino acid sequences of filovirus NP (MARV: *Marburg marburgvirus*, RAVV: *Marburg marburgvirus*, EBOV: *Zaire ebolavirus*, SUDV: *Sudan ebolavirus*, RESTV: *Reston ebolavirus*, BDBV: *Bundibugyo ebolavirus*, TAFV: *Taï Forest ebolavirus*, BOMV: *Bombali ebolavirus*, LLOV: *Lloviu cuevavirus*) were aligned with ClustalW2.1[42] and visualized with ESPript 3.0[43]. The numbering of the alignments is assigned based on the MARV NP sequence. Conserved residues are highlighted in red and similar residues are colored in red.

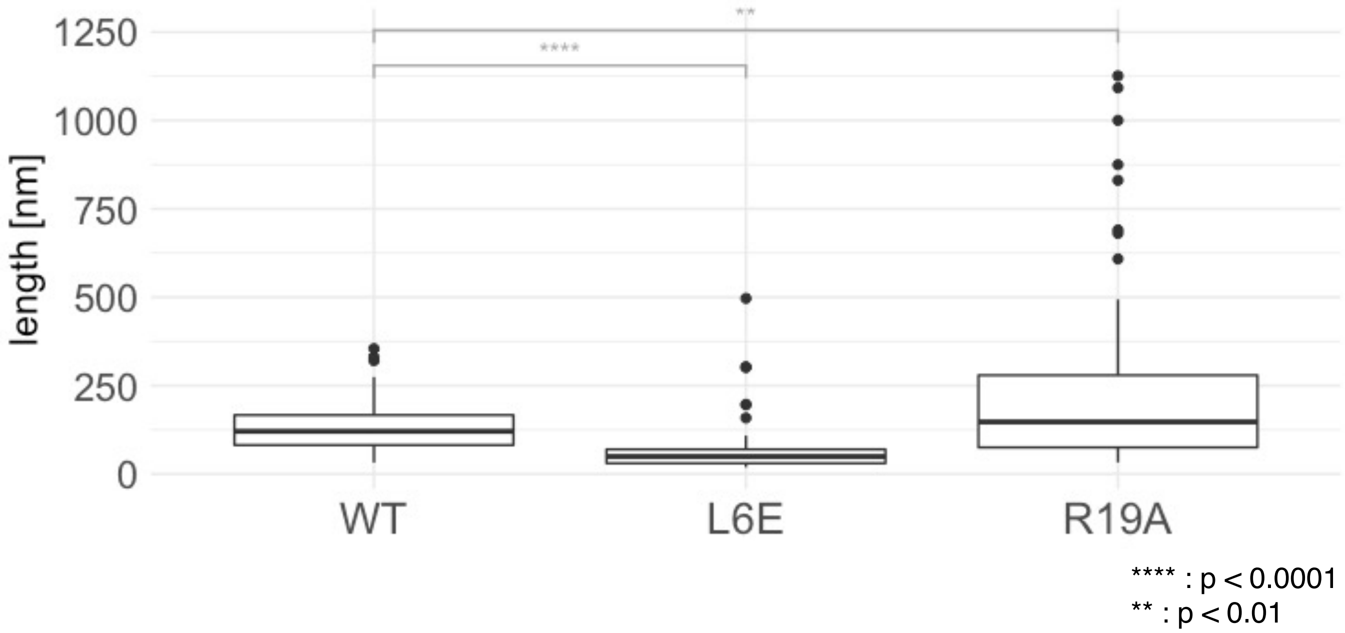

Supplementary Fig. 6: Impact of L6E and R19A mutations on the length of MARV NPs.
Length of MARV C-terminal truncated L6E and R19A mutant NPs and the C-terminal truncated wild-type (WT), evaluated by negative staining. About sixty helices were examined and the statistical significance was tested by two-sided student's t-test. (**** : $p < 0.0001$, ** : $p < 0.01$) Box-and-whiskers plots represented the maxima, 75th percentile, median, 25th percentile, and minima, with a dot indicating the missing value. Source data are provided as a Source Data file.

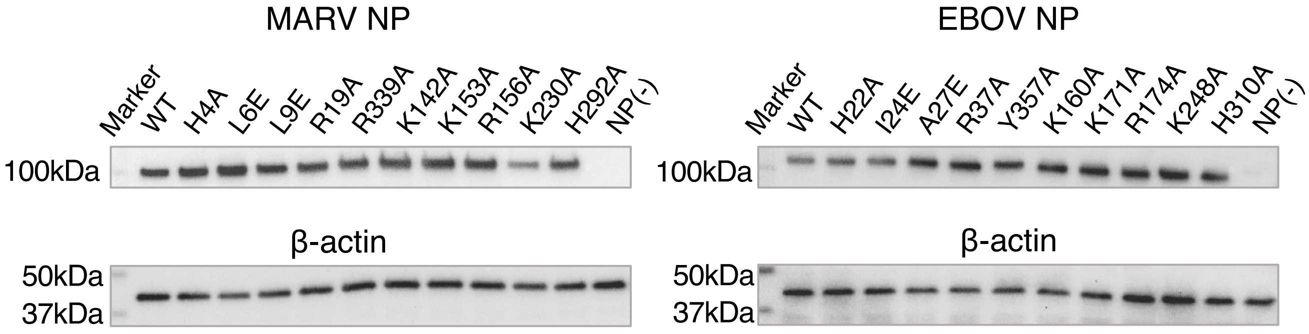

Supplementary Fig. 7: Expression levels of MARV and EBOV NP mutants in cells.
Western blot analysis of cells transfected with plasmids expressing MARV or EBOV nucleocapsid components. MARV and EBOV NP were detected using the respective specific antibodies. The experiments were performed in duplicates (n = 2).

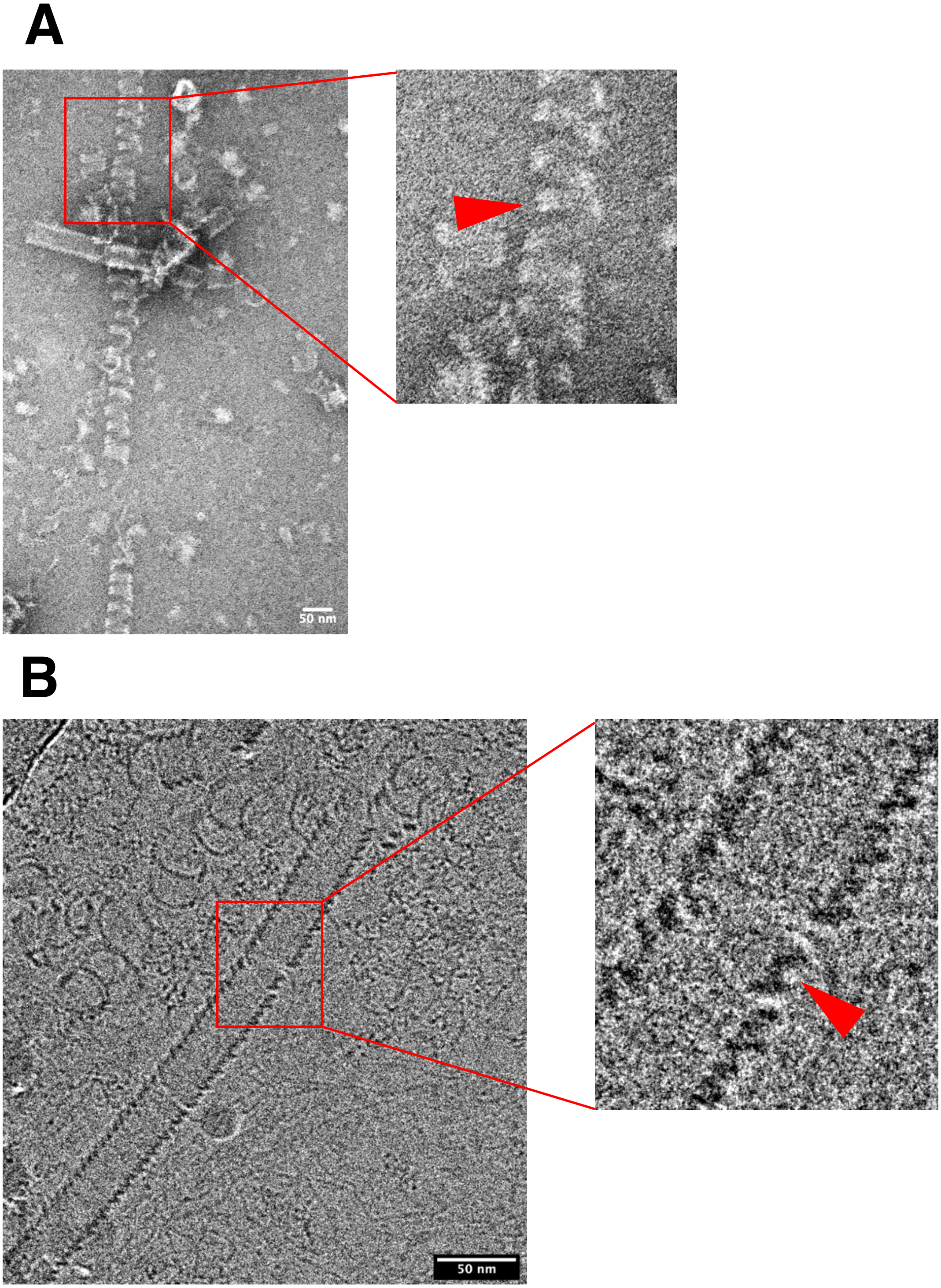

Supplementary Fig. 8: MARV NP−RNA complexes purified from HEK 293T cells.
(A) An image of the negatively stained C-terminal truncated MARV NP (1-395)-RNA complexes purified from HEK 293T cells. Red arrow indicates a double-helical structure. The experiments were performed in duplicates (n = 2).
(B) A cryo-EM image of the C-terminal truncated MARV NP (1-395)-RNA complexes purified from HEK 293T cells. Red arrow indicates a double-helical structure. The experiments were performed in duplicates (n = 2).

	Paramyxoviridae			
	Parainfluenza virus 5	Measles virus	Respiratory Syncytial Virus	Human metapneumovirus
PDB-ID	4XJN	4UFT	2WJ8	5FVC
N-terminal	C181, S191, **R195**, **K198**	**K180**, T183, **R194**	**K170**, A172, A173, **R184**, **R185**, N249	**K171**, A173, S174, V175, **R185**, **R186**, **R189**, V190, L243, N246
C-terminal	L271, A327, N346, Y350, A351, S355	G265, A267, S349, N351, F352	S313, T315, I333, G335, Y337, **R338**	G255, T257, S314, A316, I334, M337, Y338, **R339**, G340, **R341**
Surface potential	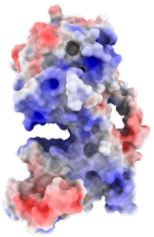	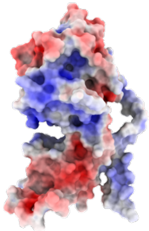	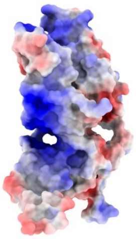	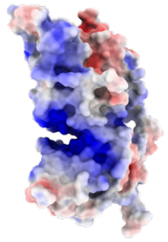
Schematic diagram of interactions with RNA	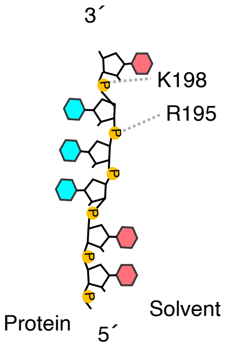	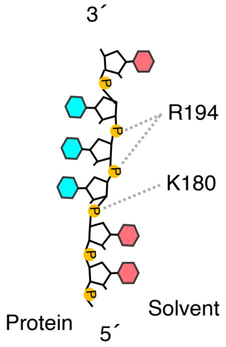	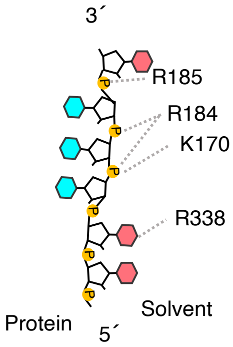	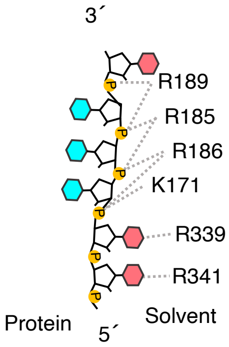

Supplementary Fig. 9: List of amino acids located near RNA strand and electrostatic surfaces on nucleoproteins of the family *Paramyxoviridae*.

Amino acids located within 3.5 Å distance from RNA strand are listed. Basic residues are highlighted in blue. None of the basic amino acids in the C-terminal lobe were oriented toward the phosphate group of the RNA (i.e., unlikely to be responsible for interaction with RNA). Electrostatic surfaces were calculated from the previously reported atomic models using Delphi web-server[40,41]. Scale ranging from -5 (red) to +5 kcal mol^{-1} e^{-1} (blue).

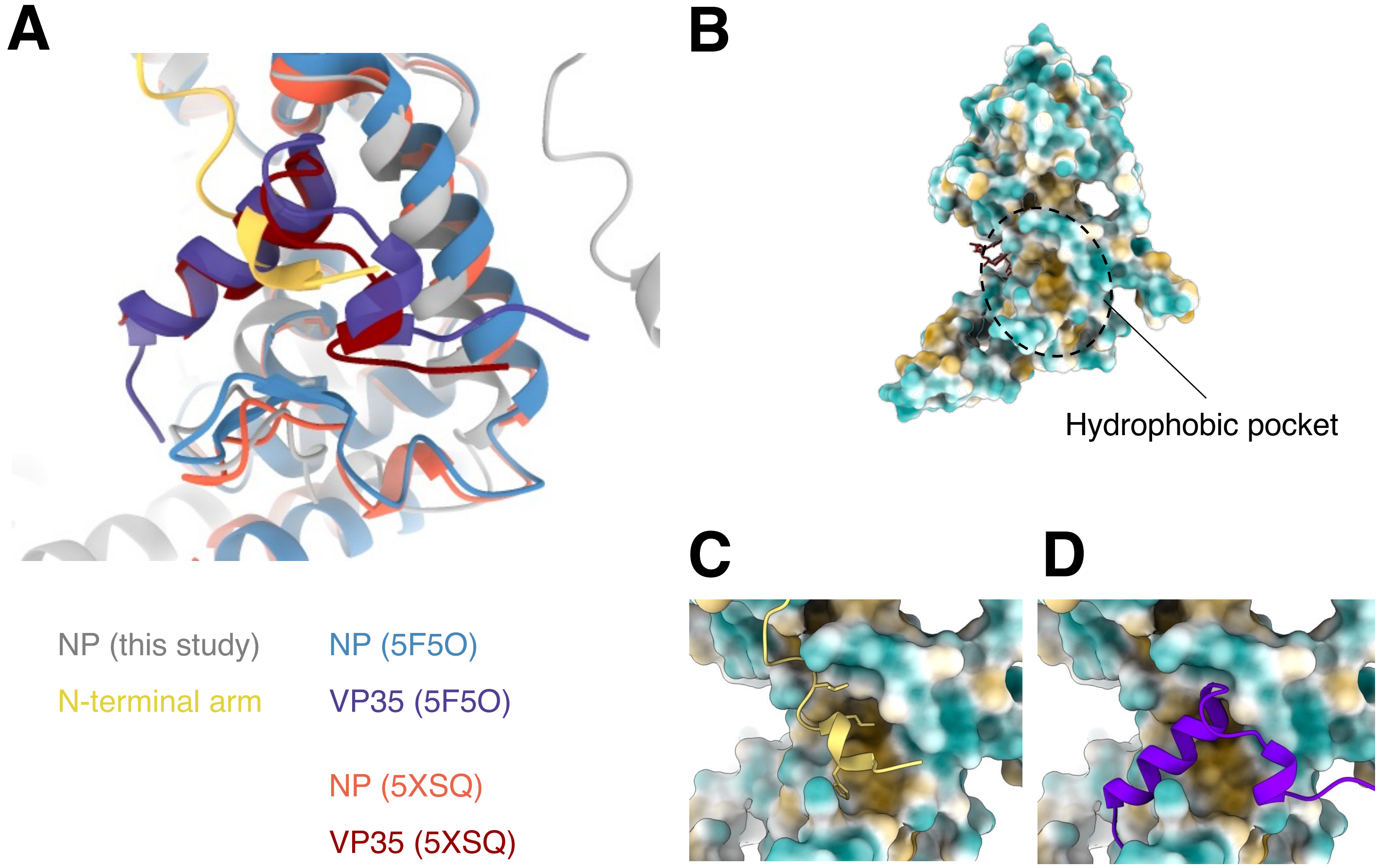

Supplementary Fig. 10: Common binding pocket in VP35 peptide and the N-terminal arm of NP.
(A) Comparison of the MARV NP structures around the binding pocket of VP35 peptide and the NP N-terminal arm. The VP35-bound RNA-free monomeric state of the NP are colored in blue and purple (PDB-ID: 5F5O), and orange and red (PDB-ID: 5XSQ). The RNA-bound oligomeric state was colored in gray and yellow (PDB-ID: 7F1M in this study).
(B) Molecular lipophilicity potential map of our structure colored in blue (hydrophilic) to orange (hydrophobic). The binding pocket of VP35 peptide and the N-terminal arm is hydrophobic.
(C) Close-up view of the N-terminal arm of its adjacent NP (shown in yellow, PDB-ID: 7F1M in this study).
(D) Close-up view of the VP35 peptide (shown in purple, PDB-ID: 5F5O) aligned relative to the whole NP structure.

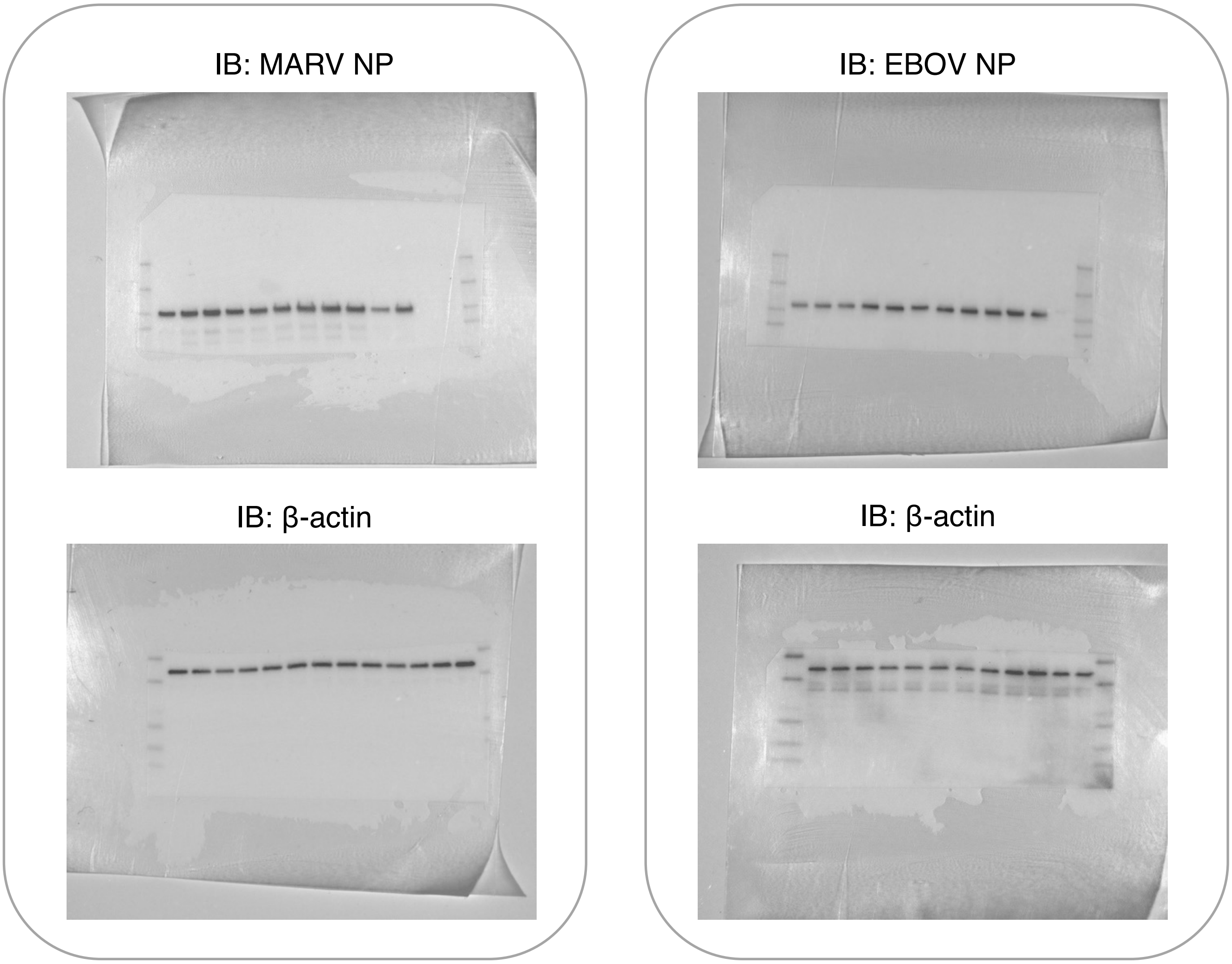

Supplementary Fig. 11: Uncropped original western blot images.

Supplementary References

40. Smith, N. *et al.* DelPhi web server v2: incorporating atomic-style geometrical figures into the computational protocol. *Bioinforma. Oxf. Engl.* **28**, 1655–1657 (2012).

41. Sarkar, S. *et al.* DelPhi Web Server: A comprehensive online suite for electrostatic calculations of biological macromolecules and their complexes. *Commun. Comput. Phys.* **13**, 269–284 (2013).

42. Larkin, M. A. *et al.* Clustal W and Clustal X version 2.0. *Bioinforma. Oxf. Engl.* **23**, 2947–2948 (2007).

43. Robert, X. & Gouet, P. Deciphering key features in protein structures with the new ENDscript server. *Nucleic Acids Res.* **42**, W320–W324 (2014).

Supplementary Table 1. The calculated MM-GBSA binding free energies (kcal mol^{-1}) of RNA (Uracil) backbone and base

URACIL<br>The order of numbering is as shown in Fig. 1e	Total	Backbone	BASE
**1**	−5.187	−3.876	−1.311
**2**	−3.003	−2.743	−0.26
**3**	−9.744	−3.957	−5.787
**4**	−5.038	−3.472	−1.565
**5**	−9.094	−8.775	−0.318
**6**	−15.876	−13.984	−1.892
**all**	−47.942	−36.807	−11.133

Supplementary Table 2. Target residues and site-specific substitutions

Mutant		Location	Contact region
MARV	EBOV		
H4A	H22A	N-terminal arm	C-terminal loop of adjacent NP
L6E	I24E	N-terminal arm	C-terminal hydrophobic pocket of adjacent NP
L9E	A27E	N-terminal arm	C-terminal hydrophobic pocket of adjacent NP
R19A	R37A	N-terminal arm	N-terminal lobe of adjacent NP
R339A	Y357A	C-terminal lobe α-helix	C-terminal lobe α-helix of adjacent NP
K142A	K160A	N-terminal lobe side of RNA cleft	RNA
K153A	K171A	N-terminal lobe side of RNA cleft	RNA
R156A	R174A	N-terminal lobe side of RNA cleft	RNA
K230A	K248A	C-terminal lobe side of RNA cleft	RNA
H292A	H310A	C-terminal lobe side of RNA cleft	RNA

Supplementary Table 3. Primers used in this study

Name	Forward or Reverse	Oligo sequence (5 to 3)	Purpose
MARV H4A	Forward	GATTTAGCCAGTTTGTTGGAGTTGGGTAC	mutation introduction
MARV H4A	Reverse	CAAACTGGCTAAATCCATGGTGGCGGCG	mutation introduction
MaV_NP_L6E_F	Forward	CACAGTGAATTGGAGTTGGGTACAAAACC	mutation introduction
MaV_NP_L6E_R	Reverse	CTCCAATTCACTGTGTAAATCCATGGTGG	mutation introduction
MaV_NP_L9E_F	Forward	TTGGAGGAGGGTACAAAACCCACTGCCCC	mutation introduction
MaV_NP_L9E_R	Reverse	TGTACCCTCCTCCAACAAACTGTGTAAATC	mutation introduction
MaV_NP_R19A_F	Forward	GCCCCTCATGTCGCAAATAAGAAAGTG	mutation introduction
MaV_NP_R19A_R	Reverse	CTTATTTGCGACATGAGGGGCAGTGGGTTTTG	mutation introduction
MaV_NP_R339A_F	Forward	CAAAGGGCACATGAACATCAGGAAATTC	mutation introduction
MaV_NP_R339A_R	Reverse	TTCATGTGCCCTTTGTAGTTTTACTTCCG	mutation introduction
MaV_NP_K142A_F	Forward	CTCCCAGCACTTGTCGTCGGAGACCGAGC	mutation introduction
MaV_NP_K142A_R	Reverse	GACAAGTGCTGGGAGGAAAAGACTGCAAAATG	mutation introduction
MaV_NP_K153A_F	Forward	ATCGAAGCGGCTTTAAGACAAGTAACAGTG	mutation introduction
MaV_NP_K153A_R	Reverse	TAAAGCCGCTTCGATACTAGCTCGGTCTC	mutation introduction
MaV_NP_R156A_F	Forward	GCTTTAGCACAAGTAACAGTGCATCAAG	mutation introduction
MaV_NP_R156A_R	Reverse	TACTTGTGCTAAAGCCTTTTCGATACTAG	mutation introduction
MaV_NP_K230A_F	Forward	ATCGTGGCAACAGTTCTCGAGTTCATCTTG	mutation introduction
MaV_NP_K230A_R	Reverse	AACTGTTGCCACGATAAGAAGTCCTGAG	mutation introduction
MaV_NP_H292A_F	Forward	CTCGAAGCAGGACTCTATCCTCAGCTTTC	mutation introduction
MaV_NP_H292A_R	Reverse	GAGTCCTGCTTCGAGGTTGTTAATCCC	mutation introduction
MaV-F-Kozac-EcoRI	Forward	CACACAGAATTCGCCGCCACCATGGATTTACACAGTTTGTTGGAGTTG	insert PCR
MaV-F-Kozac-EcoRI_H4A	Forward	CACACAGAATTCGCCGCCACCATGGATTTAGCCAGTTTGTTGGAGTTG	insert PCR
MaV-F-Kozac-EcoRI_L6E	Forward	CACACAGAATTCGCCGCCACCATGGATTTACACAGTGAATTGGAGTTG	insert PCR
MaV-F-Kozac-EcoRI-L9E	Forward	CACACAGAATTCGCCGCCACCATGGATTTACACAGTTTGTTGGAGGAG	insert PCR
MaV-R395-stop-NheI	Reverse	CACACAGCTAGCTCAAATATTGTTTTCAATTTCTGCAGCG	insert PCR
MaV_NP_full_stop_NheI_R	Reverse	CACACAGCTAGCCTACAAGTTCATAGCAACATGTCTCCTTTC	insert PCR
MaV NP seq	Forward	CACATACCCTAATCATTGGC	sequence
EboNPR174A	Forward	GTTCAAGCACAAATTCAAGTACATGCAGAGCAAGGACTGA	mutation introduction
	Reverse	AATTTGTGCTTGAACCTTCTCAAGGCAAGCCTTTTCTCCT	mutation introduction
EboNPK160A	Forward	CTTCCGGCATTGGTAGTAGGAGAAAAGGCTTGCCTTGAGA	mutation introduction
	Reverse	TACCAATGCCGGAAGGAATAGACTTGCAAAGGAGAGAAACTG	mutation introduction
EboNPK171A	Forward	CTTGAGGCAGTTCAAAGGCAAATTCAAGTACATGCAGAGCAAGGACTGATAC	mutation introduction
	Reverse	TTGAACTGCCTCAAGGCAAGCCTTTTCTCCTACTACCAA	mutation introduction
EboNPI24E	Forward	CACAAGGAGTTGACAGCAGGTCTGTCCGTTCAACAGGGGA	mutation introduction
	Reverse	TGTCAACTCCTTGTGGTAATCCATGTCAGATTCAGTGAGA	mutation introduction
EboNPR37A	Forward	ATTGTTGCACAAAGAGTCATCCCAGTGTATCAAGTAAACA	mutation introduction
	Reverse	TCTTTGTGCAACAATCCCCTGTTGAACGGACAGA	mutation introduction
EboNPH22A	Forward	GATTACGCAAAGATCTTGACAGCAGGTCTGTC	mutation introduction
	Reverse	GATCTTTGCGTAATCCATGTCAGATTCAGTGAGACTCG	mutation introduction
EboNPA27E	Forward	TTGACAGAGGGTCTGTCCGTTCAACAGGGGAT	mutation introduction
	Reverse	CAGACCCTCTGTCAAGATCTTGTGGTAATCCATGTCAGA	mutation introduction
EboNPY357A	Forward	CCAACAAGCAGCAGAGTCTCGCGAACTTGACCATCTTG	mutation introduction
	Reverse	ACTCTGCTGCTTGTTGGAGTTGCTTCTCAGCCTCAGT	mutation introduction
EboNPK248A	Forward	ATTGTCGCAACAGTACTTGATCATATCCTACAAAAGACAGAACGAGG	mutation introduction
	Reverse	TACTGTTGCGACAATCAATAAGCCTGAAAAACGAGCTTGAGC	mutation introduction
EboNPH310A	Forward	CTTGAGGCAGGTCTTTTCCCTCAACTATCGGCAATTGC	mutation introduction
	Reverse	AAGACCTGCCTCAAGATTATTTACTCCAGAAAGGTTCAAAAGTCGGGCGA	mutation introduction
EcoR1EboNPF	Forward	CACACAGAATTCGCCGCCACCATGGATTCTCGTCCTCAGAAAATCTGGATGG	insert PCR
Nhe1EboNPR	Reverse	CACACAGCTAGCTCAAGCGTCATCGTCGTCCTTGTAGTCCT	insert PCR
EboNP425F	Forward	CAACTGAAGCTAATGCCGGTCA	sequence

Supplementary Table 4. Cryo-EM data collection, refinement and validation statistics

	MARV NP−RNA complex (EMDB-31420) (PDB-ID: 7F1M)
**Data collection and processing**	
Magnification	59,000
Voltage (kV)	300
Electron exposure (e–/Å^2)	30
Defocus range (μm)	-0.5 ~ -2.5
Pixel size (Å)	1.13
Symmetry imposed	C1 helical
Helical rise (Å)	4.23
Helical rotation (°)	11.8052
Initial particle images (no.)	30,668
Final particle images (no.)	23,545
Map resolution (Å)	3.1
FSC threshold	0.143
Map resolution range (Å)	2.98 ~ 4.35
**Refinement**	
Initial model used (PDB-ID)	5Z9W
Model resolution (Å)	3.1
FSC threshold	0.143
Map sharpening *B* factor (Å^2)	-100
Model composition	
Non-hydrogen atoms	6450
Protein residues	788
Nucleotide	12
B factors (Å^2)	min/max/mean
Protein	6.92/83.43/26.05
Nucleotide	13.55/25.68/20.47
R.m.s. deviations	
Bond lengths (Å)	0.009
Bond angles (°)	1.124
Validation	
MolProbity score	1.52
Clashscore	4.98
Poor rotamers (%)	0.00
Ramachandran plot	
Favored (%)	96.17
Allowed (%)	3.83
Disallowed (%)	0
