# Supplementary material for: Structural insight into Marburg virus nucleoprotein–RNA complex formation
Source: Nat Commun. 2022 Mar 4;13:1191. doi: 10.1038/s41467-022-28802-x (PMC8897395; doi:10.1038/s41467-022-28802-x)
Supplement: Supplementary file 3 — Reporting Summary [file 41467_2022_28802_MOESM3_ESM.pdf]

nature portfolio

Corresponding author(s): Takeshi Noda

Last updated by author(s): Feb 3, 2022

Reporting Summary

Nature Portfolio wishes to improve the reproducibility of the work that we publish. This form provides structure for consistency and transparency in reporting. For further information on Nature Portfolio policies, see our Editorial Policies and the Editorial Policy Checklist.

Statistics

For all statistical analyses, confirm that the following items are present in the figure legend, table legend, main text, or Methods section.

n/a	Confirmed	
☐	☒	The exact sample size (*n*) for each experimental group/condition, given as a discrete number and unit of measurement
☐	☒	A statement on whether measurements were taken from distinct samples or whether the same sample was measured repeatedly
☐	☒	The statistical test(s) used AND whether they are one- or two-sided<br>*Only common tests should be described solely by name; describe more complex techniques in the Methods section.*
☒	☐	A description of all covariates tested
☐	☒	A description of any assumptions or corrections, such as tests of normality and adjustment for multiple comparisons
☐	☒	A full description of the statistical parameters including central tendency (e.g. means) or other basic estimates (e.g. regression coefficient) AND variation (e.g. standard deviation) or associated estimates of uncertainty (e.g. confidence intervals)
☐	☒	For null hypothesis testing, the test statistic (e.g. *F*, *t*, *r*) with confidence intervals, effect sizes, degrees of freedom and *P* value noted<br>*Give P values as exact values whenever suitable.*
☒	☐	For Bayesian analysis, information on the choice of priors and Markov chain Monte Carlo settings
☒	☐	For hierarchical and complex designs, identification of the appropriate level for tests and full reporting of outcomes
☒	☐	Estimates of effect sizes (e.g. Cohen's *d*, Pearson's *r*), indicating how they were calculated

Our web collection on statistics for biologists contains articles on many of the points above.

Software and code

Policy information about availability of computer code

Data collection	Cryo-EM data was collected automatically using the EPU software (v2) available from Thermo Fisher scientific. Negative-stained TEM images were captured using HT7700 (system software v.02.22.15.15).
Data analysis	EM image processing used RELION 3.1, GCTF (v1), PyMOL (2.4.0), Chimera (1.13.1), and ChimeraX (0.91). GCTF, Chimera, and ChimeraX are free for non-commercial use and can be downloaded. The other programs are available as open source packages. Atomic model building used the open source package COOT (0.8.9.2), PHENIX (1.18) and MolProbity (4.4), which is free for non-commercial work and can be downloaded. Negative staining EM images were analyzed by ImageJ (2.1.0 or 2.3.0). Molecular dynamics simulation used Amber18 and Ambertools18. Statistical analyses were performed by using available R packages (https://www.r-project.org/).

For manuscripts utilizing custom algorithms or software that are central to the research but not yet described in published literature, software must be made available to editors and reviewers. We strongly encourage code deposition in a community repository (e.g. GitHub). See the Nature Portfolio guidelines for submitting code & software for further information.

Data

Policy information about availability of data

All manuscripts must include a data availability statement. This statement should provide the following information, where applicable:

- Accession codes, unique identifiers, or web links for publicly available datasets
- A description of any restrictions on data availability
- For clinical datasets or third party data, please ensure that the statement adheres to our policy

The cryo-EM map of the Marburg NP-RNA complex was deposited in the Electron Microscopy Data Bank (EMDB code EMD-31420). Raw movies have been deposited in the Electron Microscopy Public Image Archive with accession codes EMPIAR-10733. The atomic coordinates were deposited in the Protein Data Bank

(PDB code 7F1M).

Field-specific reporting

Please select the one below that is the best fit for your research. If you are not sure, read the appropriate sections before making your selection.

☒ Life sciences ☐ Behavioural & social sciences ☐ Ecological, evolutionary & environmental sciences

For a reference copy of the document with all sections, see nature.com/documents/nr-reporting-summary-flat.pdf

Life sciences study design

All studies must disclose on these points even when the disclosure is negative.

Sample size	Sample sizes were determined by available electron microscopy time and density of particles on electron microscopy grids. The data size was validated to be sufficient because it could provide a high resolution 3d reconstruction of the specimen.
Data exclusions	All acquired images were analyzed and parts were later excluded as is usual for such image processing studies. Misaligned image segments were excluded from averages based on cross-correlation scores and visual analysis (described in methods section and Extended Data Table 4).
Replication	Mini-genome assay and sample preparation for negative staining were all performed three times independently. All attempts at replication were successful.
Randomization	For independent single-particle alignment in 3D refinement, datasets were split into two independent subsets which were effectively randomized. For other experiments and analyses, it was unnecessary to perform randomized experiments in the current study.
Blinding	Unlike in crystallography (e.g. cross-validated R-factor), there are no well-established procedures for blinding in cryo-EM. We used referencefree 2D classification and a featureless 3d cylinder as initial model for 3D analysis to avoid reference bias. For other experiments and analyses, it was unnecessary to perform blinded experiments in the current study.

Reporting for specific materials, systems and methods

We require information from authors about some types of materials, experimental systems and methods used in many studies. Here, indicate whether each material, system or method listed is relevant to your study. If you are not sure if a list item applies to your research, read the appropriate section before selecting a response.

Materials & experimental systems

n/a	Involved in the study	
☐	☒	Antibodies
☐	☒	Eukaryotic cell lines
☒	☐	Palaeontology and archaeology
☒	☐	Animals and other organisms
☒	☐	Human research participants
☒	☐	Clinical data
☒	☐	Dual use research of concern

Methods

n/a	Involved in the study	
☒	☐	ChIP-seq
☒	☐	Flow cytometry
☒	☐	MRI-based neuroimaging

Antibodies

Antibodies used	A rabbit polyclonal antibody for Marburg virus nucleoprotein (NP) was provided from Prof. Ayato Takada, Hokkaido University and used at a 1:10000 dilution. A rabbit polyclonal antibody for Ebola virus nucleoprotein (NP) was obtained from IBT BioServices (Cat#0301-012, Lot#1401009) and used at a 1:10000 dilution. A β-actin monoclonal antibody (mouse) was obtained from Abcam (#ab8226, Clone number mAbcam8226) and used at a 10,000-fold dilution. Anti-mouse IgG HRP antibody and anti-rabbit IgG HRP antibody were obtained from GE Healthcare (NA931 and NA 934, respectively) and were used at a 1:10000 dilution, respectively.
Validation	For commercially available antibodies used in this study, see the corresponding webpages for reference and validation. The promary antibody of Marburg virus nucleoprotein is a polyclonal rabbit antibody that has been confirmed by the supplier laboratory for use in western blotting.

Eukaryotic cell lines

Policy information about cell lines

Cell line source(s)	Human embryonic kidney (HEK) 293T cell line and Human embryonic kidney 293 Freestyle (HEK293F) cells were obtained from ATCC.

Authentication	The cell line was provided by supplier (ATCC) and not independently authenticated.
Mycoplasma contamination	The elimination of mycoplasma was confirmed by ATCC.
Commonly misidentified lines (See ICLAC register)	No commonly misidentified cell lines were used.
